# A Deep Learning Approach for Autonomous Compression Damage Identification in Fiber-Reinforced Concrete Using Piezoelectric Lead Zirconate Titanate Transducers

**DOI:** 10.3390/s24020386

**Published:** 2024-01-09

**Authors:** George M. Sapidis, Ioannis Kansizoglou, Maria C. Naoum, Nikos A. Papadopoulos, Constantin E. Chalioris

**Affiliations:** 1 Laboratory of Reinforced Concrete and Seismic Design of Structures, Structural Engineering Science Division, Civil Engineering Department, School of Engineering, Democritus University of Thrace, 67100 Xanthi, Greece; gsapidis@civil.duth.gr (G.M.S.); mnaoum@civil.duth.gr (M.C.N.); nikpapad@civil.duth.gr (N.A.P.); 2 Department of Production and Management Engineering, School of Engineering, Democritus University of Thrace, V. Sofias 12, 67132 Xanthi, Greece; ikansizo@pme.duth.gr

**Keywords:** structural health monitoring (SHM), concrete damage identification, convolutional neural network (CNN), 1-D CNN, damage classification, deep learning, electromechanical impedance (EMI), fiber-reinforced concrete (FRC), piezoelectric lead zirconate titanate (PZT)

## Abstract

Effective damage identification is paramount to evaluating safety conditions and preventing catastrophic failures of concrete structures. Although various methods have been introduced in the literature, developing robust and reliable structural health monitoring (SHM) procedures remains an open research challenge. This study proposes a new approach utilizing a 1-D convolution neural network to identify the formation of cracks from the raw electromechanical impedance (EMI) signature of externally bonded piezoelectric lead zirconate titanate (PZT) transducers. Externally bonded PZT transducers were used to determine the EMI signature of fiber-reinforced concrete specimens subjected to monotonous and repeatable compression loading. A leave-one-specimen-out cross-validation scenario was adopted for the proposed SHM approach for a stricter and more realistic validation procedure. The experimental study and the obtained results clearly demonstrate the capacity of the introduced approach to provide autonomous and reliable damage identification in a PZT-enabled SHM system, with a mean accuracy of 95.24% and a standard deviation of 5.64%.

## 1. Introduction

Reinforced concrete (RC) structures constitute the majority of civil infrastructures and dwelling buildings worldwide. During their service life, RC structures are often subjected to environmental impacts, operational loading, and seismic excitations, leading to the formation of cracking and degradation of their structural integrity. Cracking in concrete is the result of stress in the material due to its brittle nature. Initially, stress-induced cracks appear as microcracks and then merge into localized macro-cracks. Localized cracks are regarded as manifestations of damage in concrete. Adding distributed fibers in concrete is the most common technique to restrain crack propagation [1,2,3]. Fiber-reinforced concrete (FRC) exhibits elevated ductility due to the presence of discrete fibers, which arrest the crack opening and confining.

A vast array of real-time structural health monitoring (SHM) methods has been developed to promptly identify the formation of damage in brittle materials and prevent catastrophic failure. In addition, some nondestructive techniques have also been developed to diagnose and locate cracks, irregularities, and structural defects, such as ultrasonics [4,5], infrared tomography [6,7,8], acoustic emission [9,10,11], the fiber Bragg grating sensor [12], eddy currents [13], and X-ray methods [1]. However, most of the above-mentioned methods are focused on metallic structures and are rather complex for in situ application to real structures. Additionally, most SHM methods require expensive equipment with high energy operational demand.

In the field of civil engineering, piezoelectric lead zirconate titanate (PZT) is a smart material with strong piezoelectric properties, and it has been playing a progressively important role in SHM because of its fast response, high sensitivity, wide bandwidth, dual sensing and actuating functions, and low cost. The electromechanical impedance (EMI) method is based on the influence of the structure’s mechanical impedance, which is affected by structural properties, such as mass, stiffness, and damping, on the EMI signature of the PZT patch. Structural damage alters the structure’s mechanical impedance and, through the coupling characteristic, is reflected in the EMI signature of the PZT patch. Structural degradation is identified by comparing the EMI signature of the PZT patch in the examined state with respect to the initial state, which corresponds to the healthy state. A large number of existing research studies in the broader literature have examined the effectiveness of EMI-based SHM of concrete due to several degradation causes, including the corrosion of steel bars in RC elements [14,15,16,17], concrete cover loss [18], the influence of the heating time [19], artificial cracks, and mass loss [20,21]. Furthermore, real-scale members of RC structures have been examined, such as beams [22,23,24,25,26,27,28,29,30], frames [31], and joints [32,33].

The EMI method has been used in several studies to assess load-induced damage in concrete specimens. Many studies have focused on flexural-induced damage in RC and FRC beams [1,30,34,35,36]. Moreover, extensive studies have investigated concrete standard specimens under compression loading [37,38,39,40,41,42,43]. Zhang et al. investigated the EMI method’s efficacy in monitoring stiffness degradation by conducting EMI measurements simultaneously with the uniaxial compression loading increment [39]. The previous studies reveal that imposed stress fields of concrete influence the EMI measurements of externally bonded and embedded PZT patches, where embedded PZT patches are more sensitive to alterations of stress fields [1,38,41]. A series of recent studies have investigated the feasibility of using PZT patches in identifying damage to anchorage in prestressed concrete, reporting empirical equations for the quantification of prestress losses [44,45]. The detection of crack formation in concrete specimens was derived from statistical indexes, such as root mean square deviation, correlation coefficient deviation, and mean absolute percentage deviation, in the majority of previous studies [38,39,41,45].

Previous research has been conducted using integrated or automated techniques, including principal component analysis, k-means clustering, and neural networks, which have been demonstrated to provide an improved ability to identify damaged types, severity levels, and locations through EMA feature analysis [29,46,47,48]. As opposed to conventional, empirically based recognition, automated EMA features were significantly superior for resolving the damage identification challenge. The recent development of deep learning techniques based on convolutional neural networks (CNNs) has emerged as one of the most powerful tools in the SHM field, and these techniques are capable of providing higher accuracy in identifying structural damage scenarios for aluminium structures with lower computing resource consumption [49]. Furthermore, deep CNNs were developed to identify concrete cracks and assess infrastructural damage through vision-based methods [50,51].

In real-life structures, their normal service loads could not be excluded from the SHM process in determining the actual level of damage. Thus, the extracted data are affected more or less, and a recognition of the introduced impact should be determined and, if possible, eliminated. In this scope, in the context of this research work, a deep learning technique was explored in the context of EMI analysis. Pre-trained 1-D CNN models have been fine-tuned for specific EMI analysis.

At first, an overview of the proposed 1-D CNN model for damage identification through EMI signatures of the PZT patch is presented. Subsequently, the experimental acquisition of 645 EMI signatures from 10 PZT patches in 4 FRC specimens under compressive loading is conducted.

Thus, this study proposes a new deep learning approach for autonomous damage identification of FRC cylinders under monotonically and repeated loading with externally bonded PZT patches. Furthermore, this experimental project investigates a novel methodology that combines the merits of using a PZT-enabled, EMI-based technique in identifying concrete damage regardless of the medium’s stress field. Furthermore, the capability of an EMI-trained neural network to identify concrete damage through a different set of PZT patches was also examined. The success or not of the latter is of utmost importance for the transition of the proposed novel approach from the experimental stage to real-life SHM applications. Finally, the efficacy of the proposed model is examined in a leave-one-out cross-validation scenario for damage identification from EMI signatures of externally bonded PZT patches. To the authors’ best knowledge, while the leave-one-out cross-validation scenario is a more realistic validation procedure, it has yet to be used in damage identification through EMI signatures.

## 2. Methodology

### 2.1. Electromechanical Impedance Technique

Piezoelectric materials, such as PZTs, are used in the EMI technique because they possess the characteristic feature of producing surface electric charges in response to mechanical stress whilst undergoing mechanical deformation in response to an electric field. Consequently, the damage-induced change in the mechanical impedance of the structural element is reflected in the electrical impedance of an actuated PZT mounted to the structure. A structure or its structural elements are continuously monitored by measuring the impedance signature of the PZT in a predefined excitation frequency band. The variations in this signature usually indicate that structural damage has occurred. In this way, alterations in the impedance signatures of smart piezoelectric transducers indicate potential structural element damage.

In the proposed methodology for identifying potential damage to an FRC specimen, a custom-made impedance analyzer excites the PZT transducers in a predefined frequency range while capturing the corresponding signals simultaneously. This custom-made device was first developed by Providakis et al. [52], called the “Wireless impedance/Admittance Monitoring System” (WiAMS), and experimentally verified [22,26,32,40]. Applying WiAMS, the EMI-based method can be integrated into broader SHM systems to monitor concrete structures continuously. This integration facilitates real-time data collection, analysis, and decision making regarding the maintenance and repair of concrete elements. 

First, a series of measurements were conducted on the undamaged FRC specimen to record the PZTs’ signatures in the healthy condition. Subsequently, measurements were performed in the same frequency range throughout the loading process, and each measurement was labelled Pre-Peak or Post-Peak according to the structural state and the mechanical response of the specimen at the time of acquisition.

The impedance signature of a PZT transducer captures its interaction with the examined structural member. Hence, structural characteristics of the structural member manifest themselves in the PZT signature due to these interactions. Bhalla and Soh introduced the concept of effective impedance to extend the one-dimensional vibration model introduced by Liang et al. to one that is two-dimensional, thus deriving the following expression of Equation (1) for the complex impedance of the bonded or embedded PZT patch. The mechanical model is derived from the interaction between the PZT patch and the host structure. Thus, it is considered an interconnected system, where the PZT patch undergoes mechanical vibrations and can be simulated as a linearly vibrating, thin rod [53].

(1) $$Z\left(\omega \right)=\frac{\bar{V}}{\bar{I}}=R+Xj=\frac{h}{\;4L^{2}\omega j}{\left[{\bar{\varepsilon }}_{33}^{Τ}-\frac{2d_{31}^{2}{\bar{Y}}^{E}}{\left(1-v\right)}+\frac{2d_{31}^{2}{\bar{Y}}^{E}}{\left(1-\nu \right)}\left(\frac{{Ζ}_{a,eff}}{Z_{s,eff}+Z_{a,eff}}\right)\left(\frac{\tan kL}{kL}\right)\right]}^{-1},$$

Notations of Equation (1).
$\bar{V}$Harmonic alternating voltage$\bar{I}$Electric currentRResistanceXReactancehThickness of PZT patchLHalf-length of the PZT patchkSpring constant${\bar{Y}}^{E}$Complex Young’s modulus of the elasticity at constant electric field${Ζ}_{a,eff}$Effective mechanical impedance${Ζ}_{s,eff}$Effective structural impedanceωAngular frequency${\bar{\varepsilon }}_{33}^{Τ}$Complex electric permittivity at constant stress$d_{31}$Piezoelectric strain coefficientjThe imaginary unitvPoisson’s ratio

Damage developing in the examined structural member will change its mass and stiffness characteristics, thus altering the structural parameters and the effective structural impedance. Thus, according to Equation (1), any change in the PZT impedance signature is correlated with damage development in the structural member. In this regard, any changes in structural mass, stiffness, and damping caused by stress or damage can be directly identified through the alterations in the EMI signature of the PZT patch.

### 2.2. 1-D CNN for Damage Identification

The advent of artificial intelligence and especially deep learning [54] has led to the development of more efficient pattern recognition [55] and automated anomaly detection systems [56], which also inspire the current work. In this subsection, the introduced methodology, including the pre-processing of the acquired impedance signature data of PZTs, as well as the architectural design of the exploited deep learning model, is described. To begin with, owing to the well-established capacity of CNNs to extract high-level patterns in combination with their decreased complexity compared to other types of deep models, like fully-connected and recurrent neural networks [57], our method is based on a convolutional architecture. 

Furthermore, despite the existing works in the field [50,51], it has been chosen to handle the impedance signature as pure time series data, i.e., each input sample is used as a 1-D vector into the model, thus enabling us to adopt a 1-D CNN architecture that further simplifies the complexity and the required processing power of the proposed system already studied in previous works [58]. 1-D CNNs excel at learning local features in sequential data. In the case of EMI, these local features may correspond to specific impedance changes or resonances at different frequencies, which are crucial for understanding an element’s structural health and state. Moreover, 1-D CNNs can automatically learn and extract discriminative features for characterization and anomaly detection.

During the pre-processing step, a representative healthy pattern for each specimen is initially defined. In particular, given the *i*-th specimen, all of the measurements under healthy conditions and for calculating their mean hi-value are collected. Then, each measurement of the *i*-th specimen at the damage state is subtracted from the hi-value, providing the input sample *x*. The above procedure represents each damaged state of the specimen at a relative distance from its healthy one, thus excluding the specific characteristics of each specimen from its damage state vector. Finally, each sample *x* is normalized by adopting the Z-score normalization, described by:

(2) $$\hat{x}=\frac{x\;-\;\sigma }{\mu }$$

where *μ* and *σ* are the mean value and the standard deviation of *x* [59].

**Figure 1:**
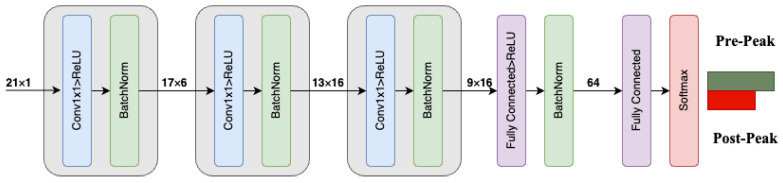
A depiction of the introduced 1-D CNN architecture.

Given the ability of CNNs to extract the most representative features from the input space, no further hand-crafted feature extraction or selection has been performed, and *x* is directly fed into the model. The architectural design of the proposed 1-D CNN is depicted in Figure 1. Furthermore, the number of kernels and their corresponding sizes for each layer are provided in Table 1, thus enabling reproduction of the model.

After each 1-D convolutional layer and the first fully connected layer, the rectified linear unit (*ReLU*) activation function is employed to address non-linearities [60] described by the following equation:

(3) $$ReLU\left(x\right)=\max\left(0,x\right)$$

where *x* is the input of the activation. Additionally, the batch normalization (BatchNorm) layer is applied after each hidden convolutional and fully connected layer, given its beneficial contribution to providing smooth training convergence and considerable performance enhancement [61]. The abovementioned layer exploits two trainable parameters, μ and σ, to estimate the mean and standard deviation of the layer’s outputs, thus normalizing the feature vector distribution before feeding them to the following layer. The output of the 1-D CNN, derived from the second and final fully connected layer, is a 1-dimensional vector $o\epsilon {\mathbb{R}}^{n_{d}}$, where nd is the number of the investigated damage states. This fully connected layer exploits the softmax activation function responsible for producing the classification result. More specifically, the output of the softmax is:

(4) $$S{\left(o\right)}_{i}=\frac{e^{o_{i}}}{\sum_{j=1}^{n_{d}}e^{o_{j}}}$$

with *S*(*o*)*_i_* denoting the softmax output of the *i*-th output neuron for *i* = 1,…, nd.

## 3. Experimental Investigation

### 3.1. Materials and Specimens

The experimental design involves casting four FRC standard cylinders of 150 mm × 300 mm (diameter × height). This study used ready-mix grade C30/37 concrete following the EN 206 specifications. A commercial type II-42.5 N Portland cement was used with coarse aggregate, fine sand, and water in a mass proportion of 1:2.8:3.3:0.56, respectively. The coarse aggregate was made from crushed limestone with a nominal particle size of up to 16 mm, while the fine aggregate was a mix of crushed limestone and natural silica sand. The fibers were directly incorporated into the freshly poured concrete at approximately 5 kg/m^3^ dosage in a randomly distributed direction, which does not affect the experimental process quality, as, on the one hand, all of the specimens have been manufactured in one batch, and, on the other hand, and most importantly, the mechanical response of the specimens was not an aspect of comparison. 

The macro-synthetic fibers used in this study are composed of polyolefin. Their shape is unique, with continuous wavy embossing to increase their bonding characteristics with concrete. The aspect ratio was 70, with a length of 50 mm and a 0.715 mm equivalent diameter. According to the manufacturer’s specifications, the mechanical characteristics of the fibers under tension were 6 GPa Young’s modulus and 430 MPa tensile strength.

The FRC mixtures were meticulously prepared and blended using a pan-type forced mixer. Initially, a pan-type mixer was employed to discharge the freshly prepared ready-mix concrete. Emphasis was given to ensuring an even distribution of fibers throughout the pan during the fiber addition process to achieve uniform fiber dispersion and enhance the flowability of the fresh FRC mix. Fibers were introduced gradually by hand in small increments during stirring to prevent the formation of clumps. Continuous stirring of the mixture guaranteed improved workability and uniform distribution of materials throughout the FRC, thus mitigating the risk of fiber segregation. In the casting phase, the prepared FRC mixture was poured into cylindrical molds and compacted using a mechanical needle vibrator to eliminate entrapped air. Careful attention was given to the pouring process and the application of continuous high-frequency vibration to fill air voids in the cylindrical formworks, thus enhancing the compactness of the FRC. The efficiency of working and compacting the FRC mixture was facilitated by adding a relatively small quantity of synthetic fibers at the consistency of the designed mix.

Specimens were cured according to ASTM C192 [62] in a moisture room at a temperature of 23 ± 1 °C and a relative humidity above 95% from the time of demolding until they were 28 days old. Additionally, specimens were kept in a vibration-free environment for 48 h from the casting time. Afterward, a grinding wheel was employed to ensure the planeness of the ends of the specimens and their perpendicularity with respect to the longitudinal axis of the cylindrical specimens.

**Figure 2:**
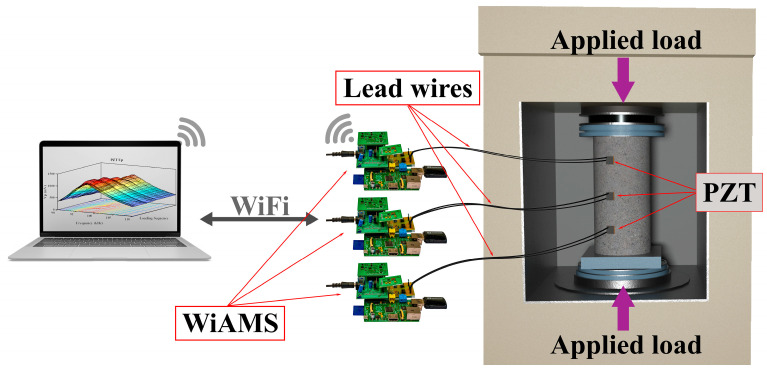
Schematic representation of the experimental setup for acquiring PZTs’ EMI signatures from FRC specimens subjected to a compression loading sequence.

Then, the PZT patches adhered to the specimen surface in predefined places, as illustrated in Figure 2. PZT Up was placed 75 mm from the top of the specimen, PZT Mid was placed in the middle of the specimen, and PZT Down was set 75 mm from the bottom of the specimen. An epoxy adhesive layer was used to bond the PZT patches on the external surface of the FRC specimen. This study used PZT patches with 20 mm × 20 mm × 5 mm dimensions, and their primary material properties are outlined in Table 2.

### 3.2. Compression Test and Data Acquisition for SHM

The experimental program involved placing four cylindrical specimens under compression loading. Two specimens were subjected to monotonic loading, while the remaining two were subjected to repeated loading. The loading test was conducted according to the ASTM C39 standard practice [63]. Therefore, a universal testing machine equipped with a displacement control function and a maximum loading capacity of 3000 kN was used to apply the loading sequence, while, simultaneously, three WiAMS devices captured the EMI signatures of the PZT patches.

**Figure 3:**
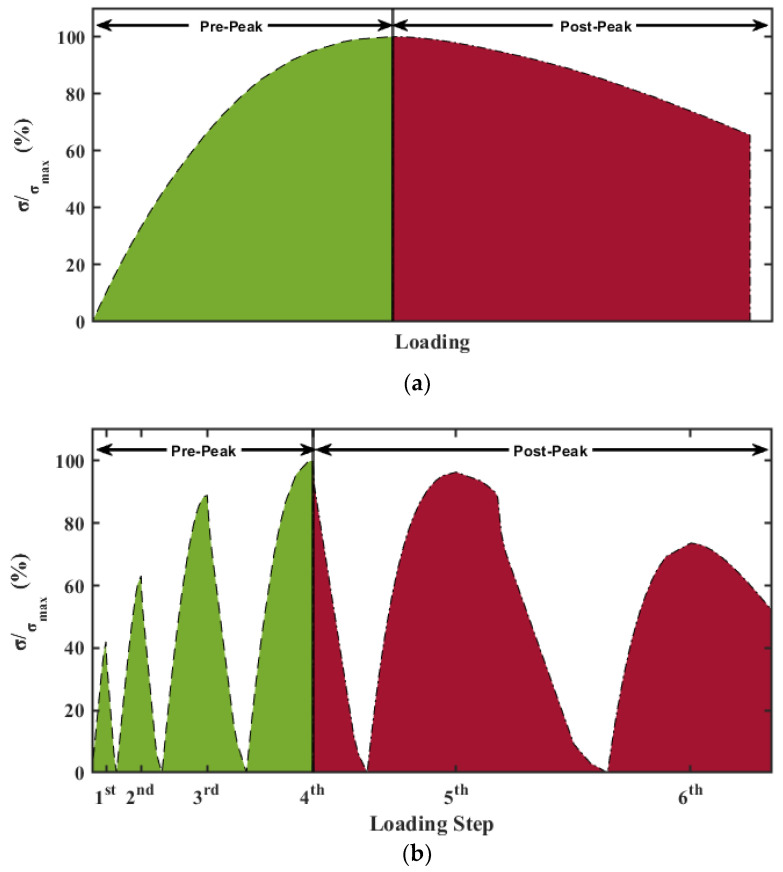
The compression loading sequence: (**a**) monotonic; (**b**) repeatable.

The loading rate was set at 1.2 mm/min for the monotonic loading test until the softening region of FRC reached 65% of the maximum stress. A series of 20 EMI measurements were collected through the WiAMS device for each PZT patch before any external loading to serve as the baseline, thus representing the EMI measurement in the healthy condition. Subsequently, EMI measurements were conducted simultaneously with the compression loading sequence and categorized into two classes. The EMI measurements, which were carried out while the FRC was in the elastoplastic deformation zone, were assigned to the Pre-Peak class, while the EMI measurements, which were carried out while the FRC was in the rapture zone, were assigned to the Post-Peak class. In Figure 3a, the elastoplastic deformation and the rapture zone are marked with green and red, respectively.

Specimens 3 and 4 were subjected to incremental repeated compressive loading (loading, unloading, reloading, etc.) using six (6) different loading steps according to their determined behavior under compressive loading. The first four loading steps were determined as a percentage of the estimated maximum strength of the specimen and corresponded to the elastoplastic region. The 5th and 6th loading steps were determined with respect to the estimated ultimate deformation and corresponded to the rupture zone. The loading sequence of repeated loading is shown in Figure 3b. During each loading step, WiAMS devices continuously captured the EMI signature of the externally bonded PZT patches throughout each step’s loading and unloading branches until the specimen reached the release condition. The labelling of EMI measurements was carried out similarly to monotonic loading. All of the EMI measurements carried out in the first three steps and the EMI measurements captured in the loading part of the 4th step were assigned in the Pre-Peak class. The EMI measurements captured in the unloading part of the 4th step and all of the EMI measurements carried out in the 5th and 6th steps were assigned in the Post-Peak class. The frequency range was set near the resonant frequency of the PZT patches, which was 90–110 kHz, with 1 kHz resolution for the PZT patches used in this study.

## 4. Results

This section describes the experimental study of the system introduced in Section 2. In particular, the evaluation protocol adopted based on the related literature to assess our method in realistic scenarios is explained. Then, the proposed 1-D CNN training setup, allowing the reader to reproduce the experimentation, is presented along with the obtained results. Furthermore, discussion and justification of the findings of our experimental study are included.

### 4.1. Evaluation Protocol and Experimental Setup

According to Section 3.2, a total of four specimens have been employed for the acquisition of SHM data. Aiming to demonstrate the generalization capacity of the proposed model, a strict and more realistic leave-one-out cross-validation scenario [64], where, in our case, the term “out” refers to each specimen, is adopted. The above validation strategy exploits all of the data related to a specific specimen as a testing set, while the training set consists of all of the remaining ones. Hence, the testing specimen remains utterly unknown to the trained model, thus simulating the realistic scenario after the deployment of the system, where all dealt specimens will also be unknown to the system. The obtained accuracy results for the testing specimen were kept and iterated the whole procedure, leaving another specimen as a testing one. Given that the proposed dataset consists of four specimens, the validation procedure has been repeated four times, ending with a four-fold cross-validation strategy [65].

Regarding the training procedure of the model depicted in Table 1, a set of hyper-parameters and training parameters that ensured competitive identification performance is defined. The well-established Adam optimizer [66] with a learning rate of 10^3^ has been employed to adopt the CNN’s weights by minimizing the cross-entropy loss. The weights initialization was realized through the Xavier-Glorot weight initialization [67]. During training, a batch size of 32 has been adopted, thereby training the model for a total of 200 epochs. After the training procedure, the best model was obtained and exploited for testing according to the recent literature [68]. The experiments were conducted on a computer device with an i5 CPU processor and an Nvidia GeForce 1060, 6 GB GPU.

### 4.2. Performance Evaluation of the Proposed CNN Model for Damage Identification of Compression Loading

**Figure 4:**
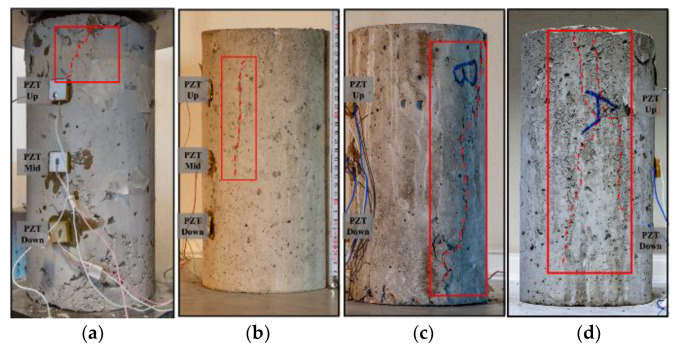
Cracking patterns after the loading sequence’s end of (**a**) specimen 1; (**b**) specimen 2; (**c**) specimen 3; (**d**) specimen 4.

The proposed CNN model was used for damage identification in FRC cylindrical specimens subjected to monotonic or repeated compression. As mentioned, specimens 1 and 2 were subjected to monotonic compressive loading, while specimens 3 and 4 were subjected to repeated compression. At this point, it is worth noting that for the specimens subjected to repeated loading, the resources of the measuring systems were not sufficient for the simultaneous recording of the measurements of all three sensors. Therefore, it was decided to examine the symmetrically mounted patches and to exclude the middle one, which, in any case, would theoretically be the one with the closest proximity to the potentially developed cracking. Figure 4 displays the specimens’ cracking patterns after the loading sequence’s end. Repeatable loading induces an extensive crack pattern in FRC specimens compared to monotonically loaded specimens, as illustrated in Figure 4.

Following the experimental procedure discussed in Section 4.1, the proposed 1-D CNN model has been tested in each specimen. In Table 3, the recognition accuracy of each PZT patch for each testing specimen is depicted. The last column summarizes the mean (*μ*) and standard deviation (*σ*) values of all PZT patches for a given specimen, while the last row demonstrates the *μ* and *σ* values obtained by each PZT. Hence, in the bottom right cell, the overall *μ* and *σ* values succeeded by the proposed 1-D CNN, considering all of the specimens and PZT patches, can be observed.

Subsequently, the confusion matrix of each PZT patch of all specimens is illustrated and discussed herein. The confusion matrices were plotted to show the fraction of correctly classified EMI measurements. The rows represent the actual class, while the columns represent the predicted class. In this way, discrepancies between the two can be identified, and the reliability of the proposed approach can be evaluated.

**Figure 5:**
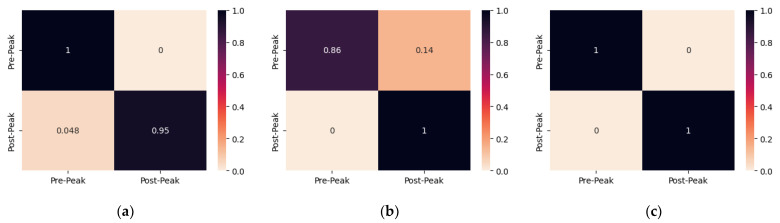
The confusion matrix of specimen 1 resulted from multi-CNN predictions for (**a**) PZT Up; (**b**) PZT Mid; (**c**) PZT Down.

The confusion matrices of the proposed CNN model for damage identification of the PZTs of specimen 1 are depicted in Figure 5. The proposed CNN model accurately identifies the state of the specimen from the EMI measurements of PZT Up, where all of the Pre-Peak measurements were classified correctly, and only the first measurement was misclassified as Pre-Peak, resulting in 95% accuracy for the Post-Peak class of PZT Up. On the contrary, the proposed model correctly identifies 86% of the Pre-Peak samples while failing to correctly classify the last Pre-Peak one and correctly recognizing all of the Post-Peak measurements of PZT Mid. The proposed CNN model succeeds with 100% classification accuracy for the EMI measurements of PZT Down.

**Figure 6:**
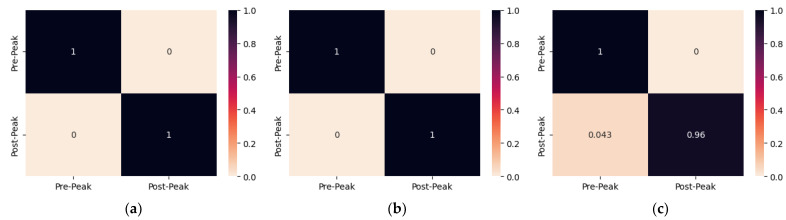
The confusion matrix of specimen 2 resulted from multi-CNN predictions for (**a**) PZT Up; (**b**) PZT Mid; (**c**) PZT Down.

Additionally, Figure 6 illustrates the confusion matrices of the proposed CNN model for damage identification of all of the PZTs of specimen 2. The proposed CNN model succeeds with 100% classification accuracy for the EMI measurements of PZT Up and PZT Mid. The critical crack of specimen 2 has a vertical orientation and is located near PZT Up and PZT Mid, as shown in Figure 4b. The introduced model also effectively identifies the specimen’s state based on the EMI measurements of PZT Down, where 100% of the Pre-Peak measurements and 96% of the Post-Peak ones were classified accurately.

**Figure 7:**
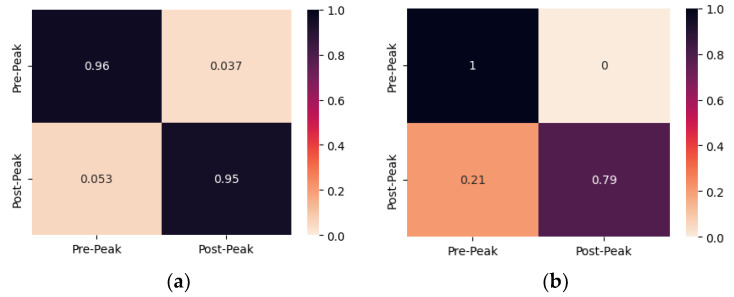
The confusion matrix of specimen 3 resulted from multi-CNN predictions for (**a**) PZT Up; (**b**) PZT Down.

Contrary to the previous specimens, specimens 3 and 4 were subjected to repeated compression loading, as discussed previously. Figure 7 depicts the confusion matrices of the proposed CNN model for damage identification of both PZTs of specimen 3. The proposed CNN model accurately classified the EMI measurements of PZT Up, where 96% and 95% of the Pre-Peak and Post-Peak EMI measurements were classified correctly. Additionally, the proposed CNN model correctly classified the Pre-Peak EMI measurements of PZT Down but misclassified 21% of Post-Peak as Pre-Peak. The direct unloading of the specimen after the formation of the critical crack in the fourth step, which formed in the upper region of the specimen, leads to the overdue crack formation in the lower region of the specimen. The eight misclassified measurements of PZT Down have been acquired between the maximum induced loading of the fourth and fifth steps, where the critical crack was still propagating towards the lowest region of the specimen. Thus, there is an apparent relationship between the location of crack formation and the PZT position, which might affect the SHM system’s overall efficacy in promptly identifying the state of the specimen.

**Figure 8:**
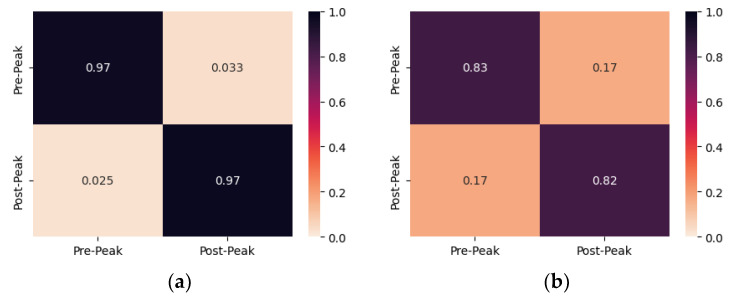
The confusion matrix of specimen 4 resulted from multi-CNN predictions for (**a**) PZT Up; (**b**) PZT Down.

Figure 8 illustrates the confusion matrices of the proposed CNN model for damage identification of both PZTs of specimen 4. The proposed CNN model accurately classified the EMI measurements of PZT Up with 97% accuracy for both specimen conditions while misclassifying one measurement of each class. On the contrary, the proposed CNN model shows the lowest accuracy in the classification of the EMI measurements of the PZT Down, where 83% and 82% of the Pre-Peak and Post-Peak were classified accurately. The five measurements that were misclassified as Post-Peak have been acquired during the maximum imposed load of the third and fourth steps. Previous studies demonstrate that the influence of the stress state of the substrate induces variation in the EMI signature, thus resulting in the misclassification of the measurements. Similarly to specimen 3, seven measurements of PZT Down, misclassified as Pre-Peak measurements, were taken between the maximum imposed load of the fourth and fifth steps when the critical crack was formed in the upper region of the specimen, thus supporting the abovementioned argument.

## 5. Conclusions

This paper proposes a 1-D CNN approach for autonomous damage identification of raw EMI signatures of externally bonded PZT transducers on FRC specimens under compression loading. The proposed approach was verified experimentally through compressive tests of FRC standard cylindrical specimens [63] under monotonic and repeated loading. The experimental program included measurements that simultaneously captured the EMI signatures of externally bonded PZT patches under different applied stress levels. The feasibility of the proposed SHM approach was validated with a leave-one-out cross-validation scenario for a stricter and more realistic validation procedure.

Based on the analyzed results, the proposed classification model processes the raw impedance signals obtained from PZT transducers and autonomously outputs the specimen’s state accurately. The accuracy of the proposed SHM approach was affected by the distance between the crack and the PZT patch locations. A prompt and efficient crack formation localization has been achieved, and the application of a dense PZT network can improve the effectiveness of the developed SHM methodology. Furthermore, 1-D CNNs are well-suited for processing sequential data, making them effective for capturing patterns and variations under the EMI-based technique.

The results also demonstrated the feasibility of the PZT-enabled EMI-based 1-D CNN approach to be used for damage monitoring of large and real-scale concrete structural members subjected to bending and shear stress under monotonic and cyclic loading. Furthermore, the proposed approach could be applied for damage monitoring of concrete specimens that could be regarded as replicates of real-life structures under compression loading in practical applications, thus potentially achieving stable and continuous monitoring of accumulative damage and, therefore, ensuring long-term serviceability.

Although the proposed classification model shows promising performance, limitations associated with the need for more extensive datasets to divide them into more structural classes have been revealed. In future work, the design of the proposed 1-D CNN model is planned to be extended to evaluate the EMI signatures of a network of PZT transducers to identify the location of the damage with further accuracy.

## Figures and Tables

**Table 1:** The architectural design of the proposed 1-D CNN.

Layer	Kernel Size	Number of Kernels
Conv1D	5	6
BatchNorm	-	-
Conv1D	5	16
BatchNorm	-	-
Conv1D	5	16
BatchNorm	-	-
Fully Connected	144	64
BatchNorm	-	-
Fully Connected	64	2
Softmax	-	-

**Table 2:** Material properties of the PZT patches.

Parameter	Value	Units
Density	7800	kgm3
Poisson ratio	0.34	-
Relative permittivity ε33Tε0	2400	-
Relative permittivity ε11Tε0	1980	-
Piezoelectric charge coefficient $d_{31}$	−210	10−12CN
Piezoelectric charge coefficient $d_{33}$	500	10−12CN
Mechanical quality factor $Q_{m}$	100	-
Dielectric loss factor	20	$10^{-3}$
Curie temperature	250	°C

**Table 3:** The obtained recognition percentages (%), as well as the mean (*μ*) and standard deviation (*σ*) values, per specimen and PZT patch.

Specimen ID	PZT Patch	Accuracy (%)	Accuracy [*μ* ± *σ*] (%)
1	Up	96.43	97.62 ± 2.06
	Mid	96.43	
	Down	100.00	
2	Up	100.00	98.81 ± 2.06
	Mid	100.00	
	Down	96.43	
3	Up	95.39	91.54 ± 5.44
	Down	87.69	
4	Up	97.14	90.00 ± 10.10
	Down	82.86	
**Overall [*μ* ± *σ*]**	Up	97.24 ± 1.98	**95.24 ± 5.64**
	Mid	98.22 ± 2.52	
	Down	91.75 ± 7.86	

## Data Availability

The data presented in this study are available upon request from the corresponding author.

## References

[1] Naoum M.C., Sapidis G.M., Papadopoulos N.A., Voutetaki M.E. (2023). An Electromechanical Impedance-Based Application of Realtime Monitoring for the Load-Induced Flexural Stress and Damage in Fiber-Reinforced Concrete. Fibers.

[2] Signorini C., Volpini V. (2021). Mechanical Performance of Fiber Reinforced Cement Composites Including Fully-Recycled Plastic Fibers. Fibers.

[3] Shafei B., Kazemian M., Dopko M., Najimi M. (2021). State-of-the-Art Review of Capabilities and Limitations of Polymer and Glass Fibers Used for Fiber-Reinforced Concrete. Materials.

[4] Altammar H., Dhingra A., Salowitz N. (2018). Ultrasonic Sensing and Actuation in Laminate Structures Using Bondline-Embedded D35 Piezoelectric Sensors. Sensors.

[5] Zhang X., Zhang L., Liu L., Huo L. (2018). Prestress Monitoring of a Steel Strand in an Anchorage Connection Using Piezoceramic Transducers and Time Reversal Method. Sensors.

[6] Halabe U.B., Vasudevan A., Klinkhachorn P., GangaRao H.V.S. (2007). Detection of Subsurface Defects in Fiber Reinforced Polymer Composite Bridge Decks Using Digital Infrared Thermography. Nondestruct. Test. Eval..

[7] Milovanović B., Gaši M., Gumbarević S. (2020). Principal Component Thermography for Defect Detection in Concrete. Sensors.

[8] Kordatos E.Z., Aggelis D.G., Matikas T.E. (2012). Monitoring mechanical damage in structural materials using complimentary NDE techniques based on thermography and acoustic emission. Comp Part B Eng..

[9] Mpalaskas A.C., Matikas T.E., Aggelis D.G., Alver N. (2021). Acoustic Emission for Evaluating the Reinforcement Effectiveness in Steel Fiber Reinforced Concrete. Appl. Sci..

[10] Zaki A., Chai H., Aggelis D., Alver N. (2015). Non-Destructive Evaluation for Corrosion Monitoring in Concrete: A Review and Capability of Acoustic Emission Technique. Sensors.

[11] Mei H., Haider M., Joseph R., Migot A., Giurgiutiu V. (2019). Recent Advances in Piezoelectric Wafer Active Sensors for Structural Health Monitoring Applications. Sensors.

[12] Wu T., Liu G., Fu S., Xing F. (2020). Recent Progress of Fiber-Optic Sensors for the Structural Health Monitoring of Civil Infrastructure. Sensors.

[13] Peng Y., Qi W., Chen Y., Mai R., Madawala U.K. (2023). Wireless Sensor Power Supply Based on Eddy Currents for Structural Health Monitoring. IEEE Trans. Ind. Electron..

[14] Hire J.H., Hosseini S., Moradi F. (2021). Optimum PZT Patch Size for Corrosion Detection in Reinforced Concrete Using the Electromechanical Impedance Technique. Sensors.

[15] Morwal T., Bansal T., Azam A., Talakokula V. (2023). Monitoring Chloride-Induced Corrosion in Metallic and Reinforced/Prestressed Concrete Structures Using Piezo Sensors-Based Electro-Mechanical Impedance Technique: A Review. Measurement.

[16] Talakokula V., Bhalla S., Gupta A. (2014). Corrosion Assessment of Reinforced Concrete Structures Based on Equivalent Structural Parameters Using Electro-Mechanical Impedance Technique. J. Intell. Mater. Syst. Struct..

[17] Ai D., Du L., Li H., Zhu H. (2022). Corrosion Damage Identification for Reinforced Concrete Beam Using Embedded Piezoelectric Transducer: Numerical Simulation. Measurement.

[18] Ai D., Zhu H., Luo H. (2016). Sensitivity of Embedded Active PZT Sensor for Concrete Structural Impact Damage Detection. Constr. Build. Mater..

[19] Ai D., Yang Z., Li H., Zhu H. (2021). Heating-Time Effect on Electromechanical Admittance of Surface-Bonded PZT Sensor for Concrete Structural Monitoring. Measurement.

[20] Ai D., Cheng J. (2023). A Deep Learning Approach for Electromechanical Impedance Based Concrete Structural Damage Quantification Using Two-Dimensional Convolutional Neural Network. Mech. Syst. Signal Process..

[21] Tseng K.K., Wang L. (2004). Smart Piezoelectric Transducers for In Situ Health Monitoring of Concrete. Smart Mater. Struct..

[22] Papadopoulos N.A., Naoum M.C., Sapidis G.M., Chalioris C.E. (2023). Cracking and Fiber Debonding Identification of Concrete Deep Beams Reinforced with C-FRP Ropes against Shear Using a Real-Time Monitoring System. Polymers.

[23] Zapris A.G., Naoum M.C., Kytinou V.K., Sapidis G.M., Chalioris C.E. (2023). Fiber Reinforced Polymer Debonding Failure Identification Using Smart Materials in Strengthened T-Shaped Reinforced Concrete Beams. Polymers.

[24] Murad Y., Abdel-Jabar H. (2021). Flexural Behavior of RC Beams Made with Electric PVC Wires and Steel Fibers. Pract. Period. Struct. Des. Constr..

[25] Bhalla S., Kaur N. (2018). Prognosis of Low-Strain Fatigue Induced Damage in Reinforced Concrete Structures Using Embedded Piezo-Transducers. Int. J. Fatigue.

[26] Kaur H., Singla S. (2022). Non-Destructive Testing to Detect Multiple Cracks in Reinforced Concrete Beam Using Electromechanical Impedance Technique. Mater. Today Proc..

[27] Kaur H., Singla S. (2023). Assessment of Reinforced Concrete Beam with Electro-Mechanical Impedance Technique Based on Piezoelectric Transducers. Arab. J. Sci. Eng..

[28] Jinesh N., Shankar K. (2019). Sub-Structural Parameter Identification Including Cracks of Beam Structure Using PZT Patch. Inter. J. Comput. Methods Eng. Sci. Mech..

[29] Perera R., Torres L., Ruiz A., Barris C., Baena M. (2019). An EMI-Based Clustering for Structural Health Monitoring of NSM FRP Strengthening Systems. Sensors.

[30] Ai D., Luo H., Wang C., Zhu H. (2018). Monitoring of the Load-Induced RC Beam Structural Tension/Compression Stress and Damage Using Piezoelectric Transducers. Eng. Struct..

[31] Haq M., Bhalla S., Naqvi T. (2022). Piezo-Impedance Based Fatigue Damage Monitoring of Restrengthened Concrete Frames. Compos. Struct..

[32] Karayannis C.G., Golias E., Naoum M.C., Chalioris C.E. (2022). Efficacy and Damage Diagnosis of Reinforced Concrete Columns and Joints Strengthened with FRP Ropes Using Piezoelectric Transducers. Sensors.

[33] Naoum M., Sapidis G., Papadopoulos N., Golias E., Chalioris C., Jędrzejewska A., Kanavaris F., Azenha M., Benboudjema F., Schlicke D. (2023). Structural Health Monitoring of Reinforced Concrete Beam-Column Joints Using Piezoelectric Transducers. International RILEM Conference on Synergising Expertise towards Sustainability and Robustness of Cement-Based Materials and Concrete Structures.

[34] Kocherla A., Subramaniam K.V.L. (2020). Stress and Damage Localization Monitoring in Fiber-Reinforced Concrete Using Surface-Mounted PZT Sensors. Meas. Sci. Technol..

[35] Kocherla A., Duddi M., Subramaniam K.V.L. (2021). Embedded PZT Sensors for Monitoring Formation and Crack Opening in Concrete Structures. Measurement.

[36] Narayanan A., Kocherla A., Subramaniam K.V.L. (2018). PZT Sensor Array for Local and Distributed Measurements of Localized Cracking in Concrete. Smart Mater. Struct..

[37] Naoum M.C., Papadopoulos N.A., Voutetaki M.E., Chalioris C.E. (2023). Structural Health Monitoring of Fiber-Reinforced Concrete Prisms with Polyolefin Macro-Fibers Using a Piezoelectric Materials Network under Various Load-Induced Stress. Buildings.

[38] Narayanan A., Subramaniam K.V.L. (2016). Sensing of Damage and Substrate Stress in Concrete Using Electro-Mechanical Impedance Measurements of Bonded PZT Patches. Smart Mater. Struct..

[39] Zhang C., Yan Q., Panda G.P., Wu W., Song G., Vipulanandan C. (2020). Real-Time Monitoring Stiffness Degradation of Hardened Cement Paste under Uniaxial Compression Loading through Piezoceramic-Based Electromechanical Impedance Method. Constr. Build. Mater..

[40] Voutetaki M.E., Naoum M.C., Papadopoulos N.A., Chalioris C.E. (2022). Cracking Diagnosis in Fiber-Reinforced Concrete with Synthetic Fibers Using Piezoelectric Transducers. Fibers.

[41] Kocherla A., Subramaniam K.V.L. (2020). Embedded Smart PZT-Based Sensor for Internal Damage Detection in Concrete under Applied Compression. Measurement.

[42] Ai D., Mo F., Han Y., Wen J. (2022). Automated Identification of Compressive Stress and Damage in Concrete Specimen Using Convolutional Neural Network Learned Electromechanical Admittance. Eng. Struct..

[43] Wang Z., Chen D., Zheng L., Huo L., Song G. (2018). Influence of Axial Load on Electromechanical Impedance (EMI) of Embedded Piezoceramic Transducers in Steel Fiber Concrete. Sensors.

[44] Perera R., Huerta M.C., Baena M., Barris C. (2023). Analysis of FRP-Strengthened Reinforced Concrete Beams Using Electromechanical Impedance Technique and Digital Image Correlation System. Sensors.

[45] Pham Q.-Q., Dang N.-L., Ta Q.-B., Kim J.-T. (2021). Optimal Localization of Smart Aggregate Sensor for Concrete Damage Monitoring in PSC Anchorage Zone. Sensors.

[46] Sevillano E., Sun R., Gil A., Perera R. (2016). Interfacial Crack-Induced Debonding Identification in FRP-Strengthened RC Beams from PZT Signatures Using Hierarchical Clustering Analysis. Compos. Part B Eng..

[47] Park S., Lee J.-J., Yun C.-B., Inman D.J. (2008). Electro-Mechanical Impedance-Based Wireless Structural Health Monitoring Using PCA-Data Compression and *k*-Means Clustering Algorithms. J. Intell. Mater. Syst. Struct..

[48] Min J., Park S., Yun C.-B., Lee C.-G., Lee C. (2012). Impedance-Based Structural Health Monitoring Incorporating Neural Network Technique for Identification of Damage Type and Severity. Eng. Struct..

[49] de Oliveira M., Monteiro A., Vieira Filho J. (2018). A New Structural Health Monitoring Strategy Based on PZT Sensors and Convolutional Neural Network. Sensors.

[50] Yu Y., Rashidi M., Samali B., Mohammadi M., Nguyen T.N., Zhou X. (2022). Crack Detection of Concrete Structures Using Deep Convolutional Neural Networks Optimized by Enhanced Chicken Swarm Algorithm. Struct. Health Monit..

[51] Lee J.Y., Sim C., Detweiler C., Barnes B. (2019). Computer-Vision Based UAV Inspection for Steel Bridge Connections. Proceedings of the Structural Health Monitoring 2019.

[52] Providakis C., Tsistrakis S., Voutetaki M., Tsompanakis J., Stavroulaki M., Agadakos J., Kampianakis E., Pentes G., Liarakos E. (2016). An Innovative Active Sensing Platform for Wireless Damage Monitoring of Concrete Structures. Curr. Smart Mater..

[53] Maurya K.K., Rawat A., Jha G. (2020). Smart Materials and Electro-Mechanical Impedance Technique: A Review. Mater. Today Proc..

[54] LeCun Y., Bengio Y., Hinton G. (2015). Deep Learning. Nature.

[55] Kansizoglou I., Bampis L., Gasteratos A. (2022). Deep Feature Space: A Geometrical Perspective. IEEE Trans. Pattern Anal. Mach. Intell..

[56] Pang G., Shen C., Cao L., Hengel A.V.D. (2022). Deep Learning for Anomaly Detection: A Review. ACM Comput. Surv..

[57] Kansizoglou I., Misirlis E., Tsintotas K., Gasteratos A. (2022). Continuous Emotion Recognition for Long-Term Behavior Modeling through Recurrent Neural Networks. Technologies.

[58] Jimenez-Guarneros M., Morales-Perez C., Rangel-Magdaleno J.D.J. (2022). Diagnostic of Combined Mechanical and Electrical Faults in ASD-Powered Induction Motor Using MODWT and a Lightweight 1-D CNN. IEEE Trans. Ind. Inform..

[59] Cheadle C., Vawter M.P., Freed W.J., Becker K.G. (2003). Analysis of Microarray Data Using Z Score Transformation. J. Mol. Diagn..

[60] Agarap A.F. (2018). Deep Learning Using Rectified Linear Units (ReLU). arXiv.

[61] Ioffe S., Szegedy C. (2015). Batch Normalization: Accelerating Deep Network Training by Reducing Internal Covariate Shift. arXiv.

[62] (2004). Practice for Making and Curing Concrete Test Specimens in the Laboratory.

[63] (2004). Standard Test Method for Compressive Strength of Cylindrical Concrete Specimens.

[64] Kearns M., Ron D. (1997). Algorithmic Stability and Sanity-Check Bounds for Leave-One-out Cross-Validation. Proceedings of the Tenth Annual Conference on Computational Learning Theory—COLT’97.

[65] Wong T.-T. (2015). Performance Evaluation of Classification Algorithms by K-Fold and Leave-One-out Cross Validation. Pattern Recognit..

[66] Kingma D.P., Ba J. (2014). Adam: A Method for Stochastic Optimization. arXiv.

[67] Glorot X., Bengio Y., Teh Y.W., Titterington M. (2010). Understanding the Difficulty of Training Deep Feedforward Neural Networks. Proceedings of the Proceedings of the Thirteenth International Conference on Artificial Intelligence and Statistics, Chia Laguna Resort.

[68] Oikonomou K.M., Kansizoglou I., Gasteratos A. (2023). A Hybrid Reinforcement Learning Approach with a Spiking Actor Network for Efficient Robotic Arm Target Reaching. IEEE Robot. Autom. Lett..

